# Social media and Dentistry: ethical and legal aspects

**DOI:** 10.1590/2177-6709.24.6.080-089.sar

**Published:** 2019

**Authors:** Alexandre Henrique de Melo Simplício

**Affiliations:** 1 São Paulo State University, School of Dentistry of Araraquara, Araraquara, Brazil.; 2 Federal University of Piauí, School of Dentistry, Graduate Program in Orthodontics, Teresina, Brazil.

**Keywords:** Social media, Ethics, Forensic Dentistry

## Abstract

**Introduction:**

In a saturated market with an over-supply of undergraduate and graduate programs, social media have become attractive means of advertising in Dentistry. However, posts frequently contain ethical violations and lead to service commodification, and their contents are often in disagreement with the Code of Consumer Protection.

**Objective:**

This article, which focuses on ethical and legal developments, contributes to the discussion and elucidation of questions associated with advertising that uses clinical images and photographs of patients in social media for commercial purposes and self-promotion.

**Conclusion:**

Social media and networks are valuable tools of dissemination and exchange of information because of their flexibility, democratic character and low cost. However, their abusive or misleading use, as well as the ethical and legal violations associated with the difficulty in controlling their use, may lead to serious damages and unfavorable court decisions.

## RESUMO

**Introdução:**

diante de um mercado saturado e com excesso de oferta de cursos de graduação e pós-graduação, as mídias sociais tornaram-se um atrativo veículo para publicidade em Odontologia. Entretanto, são frequentes as postagens com violações éticas, mercantilização e conteúdo em desacordo com o Código de Defesa do Consumidor.

**Objetivo:**

o presente artigo tem como objetivo contribuir para a discussão e elucidação das questões relativas à publicação de imagens clínicas e de pacientes nas mídias sociais, com o intuito comercial e autopromoção, enfocando os desdobramentos éticos e legais.

**Conclusão:**

as mídias/redes sociais são valiosas ferramentas de divulgação e troca de informação, por sua agilidade, caráter democrático e baixo custo. Contudo, sua utilização de forma abusiva e enganosa, com infrações éticas e legais, associada à dificuldade de fiscalização, pode trazer sérios prejuízos, resultando em jurisprudências desfavoráveis.

## INTRODUCTION

The year of 2019 had a rough beginning for dentists in Brazil. On January 29, the President of the Federal Council of Dentistry (CFO) prepared a set of five *ad referendum* Resolutions to be duly approved. As they dealt with polemic topics, reactions were immediate. While some dentists celebrated the initiative, a considerable number protested against the Resolutions by pointing out incoherent issues, contradictions and non-conformities to laws and regulations.

Specifically, Resolution 196/2019^1^ changed the Code of Ethics for Dentists (CEO) in force at the time, lifting the ban on the publication of selfies of dentists, with or without their patients, as well as allowing the publication of pre- and post-treatment photographs of dental procedures, euphemistically called diagnosis/treatment completion photographs.

In the scope of the Resolutions discussed, there was a clear motivation for changes: social media have gained extraordinary expression and repercussion as means to disseminate information about Dentistry. Therefore, such changes had their origin and purpose directed to posts in social media and networks.

What are the medium- and long-term consequences of these changes for the dental labor market? Has this measure followed the formal procedures? Have ethical and legal precepts been compromised? Are there risks for dentists? Are there risks for patients? Do these practices increase the importance assigned do Dentistry? This article contributes to the discussion and elucidation of these questions.

## DENTAL LABOR MARKET

Brazil currently has 314,818 professionally active dentists^2^ with a rather uneven distribution. Only three states in the Southeastern region have 50.5% of all dentists: São Paulo - 28.7%; Minas Gerais 11.7%; and Rio de Janeiro - 10.1%. When we consider the currently estimated Brazilian population of 209,598,123 inhabitants^3^, we have a ratio of 665 inhabitants per dentist. Although the World Health Organization (WHO) has never established an ideal ratio^4^, studies in the literature often cite WHO as the source of a 1,500:1 ratio as a reference. The state of São Paulo has reached 507:1. The whole state of Piauí has 1,007:1, but if we analyze only its capital city, Teresina, we find 411:1^2^. The current distribution, therefore, indicates saturation and concentration in the largest urban centers.

The growing rate of this ratio has been alarming, as in 2010, there were 219,575 dentists. That means that in a little more than eight years, there was an increase of 95,243 dentists in the labor market, at a growth rate of 43.4%. When we consider that the yearly population growth rate for the Brazilian population is an average of 1.8%, we realize that we face rather unfavorable circumstances. This is a direct consequence of the indiscriminate opening of new undergraduate courses. In 2015, there were 220 Schools of Dentistry; today, there are 412, with an increase of 87% of the number of schools^2^ in less than four years.

As there is no planning or technical criteria for opening new schools, as well as no efficient control, the labor market in Dentistry faces the paradox of, at the same time, having areas with an overpopulation of dentists, while other regions have practically no dentists. Most dentists are young women who have graduated from private schools^5^.

This picture has the deleterious ingredients necessary to promote unfair competition, the use of improper irregular advertising and the vilification of dental procedures. There are frequent examples and reports of practices that point to serious deficiencies in education in the areas of Ethics, Legislation, Marketing and Management applied to Dentistry^6^. Unfortunately, we cannot rule out the existence of possible cases of character flaws or bad faith.

## ONLINE SOCIAL MEDIA AND NETWORKS

The production and dissemination of information and knowledge have always played a revolutionary role in the history of humanity. One example is the invention of the movable-type printing press by Gutenberg, in 1450. At that time, the estimated number of books copied by hand by monks in Europe was about 30,000. In 1500, in contrast, 15 million books had already been printed in the 250 presses distributed in several countries of that continent. This number would jump to 50 million around 1550^7^. The world would never be the same again: the popularization of books made access to knowledge democratic, and substantially accelerated scientific and cultural development. Other landmarks followed in the field of communications, such as the invention of the telegraph, telephone, radio and TV, culminating in the Internet, responsible for a current revolution, at a scale never seen before.

“Media” refers to any instrument or means of social communications. Therefore, social media and networks, which existed long before the Internet, are tools or strategies to facilitate relationships and communications between groups of people with similar interests. Global access to the Internet became possible in the end of the 1990s. In 1996, 66% of all Internet users were Americans; in 2016, 89% were from other countries^8^. Such increase has resulted in the development of online social media and networks, a phenomenon of communications that also has behavioral, economic and political repercussions. 

Online social media had an initial focus on large-scale, unidirectional dissemination of information by means of portals, sites and personal pages. Online social networks have become, in addition to means to establish interpersonal relationships, interactive platforms that count on a large variety of additional services. The first online social network that followed the model seen today was Friendster (2002). Since then, several new ones have been created, and a number of others have disappeared, such as Orkut (2004-2014). The most popular today are Facebook (2004, which opened to the public only in 2006), YouTube (2005), Twitter (2006), WhatsApp (2009) and Instagram (2011)^9^ ^,^ ^10^.

Brazil is the leader in time of access to social media in Latin America. The multiplatform audience of social networks in Brazil is larger than the sum of those of Argentina, Mexico, Colombia and Chile. The use of applications in smartphones is predominant (88%), and there is a progressive decline of the use of desktops. Facebook and WhatsApp are the leaders in time of access for all age groups. Instagram, which had the greatest growth in recent years, YouTube and Twitter have a younger audience^8^ ^,^ ^9^.

Media and social networks are, therefore, high impact instruments in the formation of contemporary opinions. They stand out because of their flexibility, wide range, ease of use and low cost, when compared with traditional media. They have become greatly efficient and influential tools for advertising and marketing strategies. A great challenge today is to find tools with that same flexibility and efficiency to ensure their regulation and legal control, as they have a libertarian and, in same cases, even anarchic character.

The sociologist Sigmund Bauman has brilliantly described some “side effects” that may be a consequence of the excessive use of these means. *“Social media don’t teach us to dialogue because it is so easy to avoid controversy… But most people use social media not to unite, not to open their horizons wider, but on the contrary, to cut themselves a comfort zone where the only sounds they hear are the echoes of their own voice, where the only things they see are the reflections of their own face. Social media are very useful, they provide pleasure, but they are a trap.”* ^*11*^

## NORMS AND LAWS THAT REGULATE MARKETING IN DENTISTRY

In a utopian world, where there would be only honest and ethical people, formal norms and laws would be dispensable. Unfortunately, reality demands the establishment of rules that protect against acts or omissions that may produce physical, material or moral damage. In the specific case of announcements, marketing and advertising in Dentistry, dentists should follow objective criteria, whose purpose should be to restrain unfair competition and commodification and to protect patients, the vulnerable party in the relationship between service providers and consumers. As the ignorance of norms and laws is not admissible as a defense argument, all professionals in Dentistry must study and understand them.

In reference to advertising, we draw attention to two Brazilian federal laws and one CFO Resolution: Law 5081/66^12^, which governs the Dental profession; Law 8078/90^13^, also known as the Code of Consumer Protection; and CFO Resolution 118/12^14^, the Code of Dental Ethics currently in force. 

Section 7 of Law 5081/66 establishes that dentists shall not “publicly exhibit dental work”, “offer consultation by mail, radio, TV or similar media”, “announce price of services, payment forms and other forms of commercial transactions that may be construed as unfair competition”^12^. As it is a Federal Law, its contents can only be amended after laws are passed in the National Congress.

Dentists are classified as service providers according to the Brazilian Code of Consumer Protection (CDC); consequently, they have a contract with their patients, even if not formalized in a written document. Such implicit contracts define mutual rights and obligations; however, when in doubt, their interpretation should be in favor of the patient. One of dentists’ obligations and patients’ rights refer to information: patients have the right to receive clear information, in accessible language, about the procedures to be performed, treatment options, treatment objectives, benefits, possible risks, costs and estimated time to completion.

The CDC guarantees that consumers will be protected against the following: (1) Subliminal advertising, that is, patients should identify that something is an ad easily and immediately; (2) Deceptive advertising, which may mislead consumers about the nature, characteristics, quality, quantity, properties, origin, price and any other characteristic of products and services; and (3) Abusive advertising, which may mislead consumers to behave in a way that may be harmful or dangerous for their own health or safety by, for example, convincing them to undergo a certain treatment that is unnecessary or potentially harmful. It is important to note that, once dentists have been convicted for such practices, they may even have to serve a prison term of six months to one year, as well as to pay fines.

The Code of Ethics for Dentists (CEO) has a chapter specifically about announcements, advertising and marketing (Chapter XVI). Objectively, this Chapter describes what must be included, what is optional (Box 1) and what is forbidden in the content of advertising messages (Box 2). All advertising, including posts in social networks, should include the name of the dentist, registration number in the Regional Council of Dentistry (CRO), as well as the indication that it refers to a dentist. In case it is a legal entity, the name and registration number of the dentist responsible for it should be included. 

**Box 1:** Chapter XVI of the Code of Ethics for Dentists - Obligatory and optional information in advertising and marketing.

CHAPTER XVI - OF COMMERCIALS, ADVERTISING AND MARKETING
Section 41. Communications and dissemination of information in Dentistry shall follow the provisions made in this Code.
Paragraph 1. Dental prosthesis technicians, oral health technicians, dental prosthesis assistants and dental prosthesis laboratories shall not produce commercials, advertisements and marketing actions directed to the public in general.
Paragraph 2. The workers listed in Paragraph 1, except oral health assistants, may post advertisements in journals, newspapers or specialized brochures, as long as they are directed to dentists and include their own name or the laboratory name, the service’s technical manager, and the number of registration in the Regional Council of Dentistry.
Paragraph 3. Dental prosthesis laboratories shall exhibit, in a place of easy visualization to the public, the information provided by the Regional Council of Dentistry about the legislation that restricts the direct provision of services to patients.
Section 42. Ads, commercials and marketing actions may be included in any means of communications as long as the provisions of this Code are followed.
Section 43. All communications and advertising of information shall obligatorily have the name and registration number of the person or business, as well as the name used for the dental profession and the other regulated auxiliary occupations. In the case of businesses, there should also be the name and registration number of the technical manager.
Paragraph 1 In addition, the following may also be included in communications and advertising actions:
I - areas of specialization, treatment procedures and techniques, as long as preceded by the name of the specialty registered in the Regional Council or of the professional qualification as a dentist. Areas of work and the procedures adopted in the specialties recognized by the Federal council;
II - specialties of registration of the dentists in the Regional Council;
III - graduate and teaching degrees in the professional area;
IV - address, phone, fax, electronic address, working hours, health insurance programs, agreements, home and hospital care;
V - graphic logo and logotype; and
VI - the title “general dentist” for dentists that work in Dentistry.
Paragraph 2. Businesses, when they refer to or illustrate specialties, should include a professional registered in the Regional Council for the specialties advertised. A list of the professionals and their qualifications should be available to the public and, in case of general dentists, they should be listed together with their areas of work resulting from the knowledge acquired in undergraduate or graduate programs.

**Box 2:** Chapter XVI of CEO - Ethical violations in advertising and marketing.

CHAPTER XVI - OF ANNOUNCEMENTS, ADVERTISING AND MARKETING
Section 44. The following are ethical violations:
I - to use deceptive or abusive advertising and marketing, which includes the use of pre- and post-treatment photographs, prices, free services, payment plans, or any other forms of commodification of Dentistry, or that in any way goes against the provisions of this Code;
II - to announce or advertise degrees, qualifications, specialties that are not held, or are not registered in or accredited by the Federal Council.
III - to advertise or announce techniques, treatments or areas of work that have not been scientifically proved, neither facilities or equipment that have not obtained a valid registration with regulatory agencies;
IV - to criticize techniques used by other dentists as inadequate or outdated;
V - to offer appointments, diagnoses or treatment prescriptions, or to publish clinical outcomes in any means of communications, as well as to let their personal participation in the publication of dental topics lose its exclusive explanatory and educational character for the community;
VI - to publish name, address or any other information that identifies patients, unless they or their guardians have given their free and informed consent for that, and as long as the publication does not have the purpose of self-promotion or benefit of the dentists themselves or the dental service company, in agreement with the provisions of this Code;
VII - to gain patients by offering or allowing the offer of services by means of false, irregular, illicit or immoral ads, with the purpose to attract patients, or to perform other acts that characterize unfair competition or vilification of the profession, particularly the use of the word “popular”;
VIII - to induce public opinion to believe that a field of clinical work is restricted to some dentists;
IX - to offer free services with the purpose of self-promotion, or to promote campaigns offering favors that characterize *quid pro quo*;
X- to announce professional services as a prize in competitions and contests of any nature or as a means to acquire other goods through the use of the services provided;
XI - to promote environmental pollution directly or indirectly by advertising or marketing;
XII - to use advertising artifices to gain customers in the public, particularly by using photographs taken before, during and after treatment or images of dental procedures;
XIII - to participate in group buying programs offering services in means of communications; and to announce and offer dental services for commercial purposes or to gain patients by using discount cards, discount programs, direct mailing via the Internet, promotional or group buying sites, active telemarketing to the population in general, promotional stands, portable boom boxes or car stereo systems, human billboards and any other means that characterize unfair competition and professional vilification.

The following information may be used in advertising and marketing: area of expertise, treatment procedures and techniques, specialties for which the dentist is registered in the CRO; graduate degrees and teaching positions in Dentistry; address, phone, electronic mail address, working hours, health insurance programs, service agreements, home and hospital care; graphic logo and logotype.

Finally, the Code lists ethical violations in this field, such as: announcing prices and payment plans, as well as offering free services, promotions and other practices that may characterize the commodification of the profession. In terms of image exposure, the CEO prohibits the following: to publish clinical results in any means of mass communications; to publish name, address or any other information that identifies patients with the purpose of self-promotion; and to use images or photographs taken before, during and after dental procedures to gain customers.

In Brazil, laws are popularly classified according to whether they are enforced or not. Some people assign themselves the right not to follow norms simply because they allegedly do not agree with them or find them obsolete. We have seen these behaviors in relation to the regulations of advertising in Dentistry. According to law, for example, people in Brazil who are in favor of abortion have the right to express that, present their reasons, defend changes in Brazilian law, but never practice it in Brazil, where it is forbidden by law. Expression is free, but practice remains a crime until society, by means of approval of proper amendments, changes legislation.

## CFO RESOLUTION 196/2019

On January 29, 2019, the CFO introduced Resolution 196/2019, which significantly changed Chapter XVI of the Code of Ethics for Dentists (CEO), to be approved by the Plenary Session. This change was ratified at an extraordinary meeting on February 21. Regardless of its contents, these procedures infringed CFO Bylaws^15^. According to these Bylaws, the President is the only person entitled to present *ad referendum* decisions made by the Board of Directors and Plenary Session when, due to their urgent status or importance, these decisions demand immediate action (Section 53, subsection XXIII), which did not apply to the case of Resolution 196/19.

The same CFO Bylaws determine that final CEO changes should only be approved during a Joint Meeting, and not by the President or the Plenary Session (Section 10, subsection I). The Joint Meeting brings together effective and alternate members of the Plenary Session, as well as all Presidents of the Regional Councils. Therefore, Resolution 196/19 may be nullified.

The CEO has been amended and updated periodically since its first edition in 1971. However, since 1991, such amendments have to be made during the Brazilian Conferences on Ethics for Dentists (CONEO). CONEO are preceded by open meetings held in the Regional Councils and bring together all dental community for full discussions of the topics to be updated, revoked or included in the new CEO. After discussions, delegates are elected directly to be part of the CONEO Plenary Session, which is responsible for writing and approving, in an open democratic vote, the guiding norms for dental practices. This procedure is called Ethics Consensus, because it effectively represents the majority opinion.

 If the CEO has to be changed to adapt to the new reality of social media and networks, changes should be the result of a full discussion, with opportunity for the analysis of different opinions. Final decisions should reflect the consensus on what is better for the whole of the dental profession and patients, not only the interests of a group, particularly if the purpose is to obtain self-promotion and individual financial gains.

## PUBLICATION OF CLINICAL IMAGES AND ITS CONSEQUENCES

Those that defend the free use of clinical images, particularly the well-know photographs taken before, during and after treatments, often use the argument of freedom of expression and information. This important individual right is a constitutional guarantee that should be preserved, as long as it is used for the common good and guided by respect and dignity. In other words, my rights end where the rights of others begin. Therefore, there is no absolute right, and individual freedom of expression should respect ethical and moral limits to ensure the prevalence and preservation of the common good and good social interactions.

The publication of clinical images for laypeople, may, in fact, have an educational role, which is beneficial to the population in general. However, what we often see in social networks are posts of clinical procedures whose purpose is to promote that individual dentist and gain customers, which characterizes these posts as advertising and marketing. The current market, with its saturation and cutthroat competition, has precipitated the use of these practices. 

A critical evaluation of postings in Facebook and Instagram, using the CEO norms as criteria, shows that most do not follow the regulations. The most frequent violation is the use of subliminal advertising, with no clear indication that it is a commercial message and no name and registration number of the dentist^16^. 

This type of practice should be analyzed in more detail. In postings of pre- and post-treatment photographs, clinical cases have to be previously selected. Do they represent the outcome of most cases treated by that dentist, or the best outcome obtained in a much larger group? Is this ethical and honest, or may it induce the layperson to misinterpret the information? Are the perfect cases accompanied by information about contraindications and inherent risks? According to the Code of Consumer Protection, the answers to these questions should be clear for the patients.

 There are also consequences in the characterization of the type of dentists’ legal obligations. Patients see photographs of clinical procedures, are “seduced” by them and have, then, the right to demand the outcome exhibited, which is a highly unfavorable demand on the dentist. When the obligation of providing a certain outcome is established, this outcome becomes the dentist’s objective responsibility. That means that any time the patient challenges the treatment outcomes, or claims damages in court, there is a reversal of the burden of proof, and the dentist will have to produce evidence that there was no error. On the one hand, this is a risk taken by individual dentists, and they have to face possible punishment. On the other hand, there is damage to all the dental class, as it establishes unfavorable precedents for future legal actions.

Reversal of burden of proof in court actions significantly reduces the chances of acquittal of a dentist^17^. In the eagerness to achieve popularity in social networks and gain customers, and thinking only in short-term results and greater financial gains, dentists are arming patients against themselves and harming the whole dental class.

The practice of Dentistry, particularly Orthodontics should be seen as an obligation of means, because of the biological variations among patients, as well as of the several external factors that may affect the course of treatments. That means that dentists should be committed to the application of all possible means available, diligently and carefully, without, however, guaranteeing a specific outcome. In these cases, responsibility is subjective, and the burden of proof lies with the person who lays charges, who, in this instance, would be the patient. Court interpretations have not been uniform in terms of the nature of dentists’ legal obligations^17^. However, case law seems to tend to the consolidation of obligation of results, because of the many cases involving clinical photographs published in social networks. 

**Figure 1:**
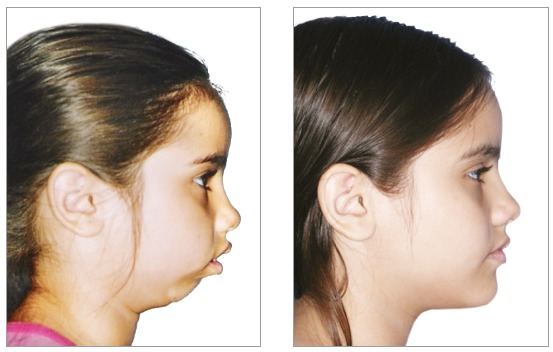
Pre- and post-treatment images of a case allegedly treated using functional orthopedics.

**Figure 2:**
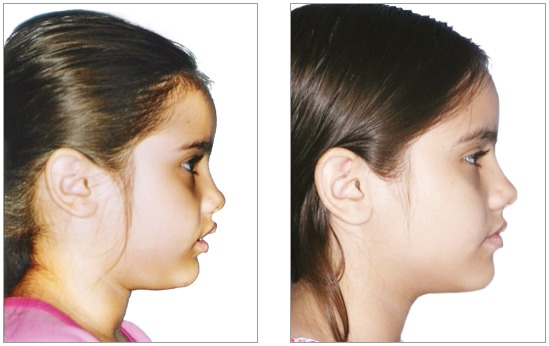
Actual pre- and post-treatment photographs of case in Figure 1; treatment using functional orthopedics.

**Figure 3:**
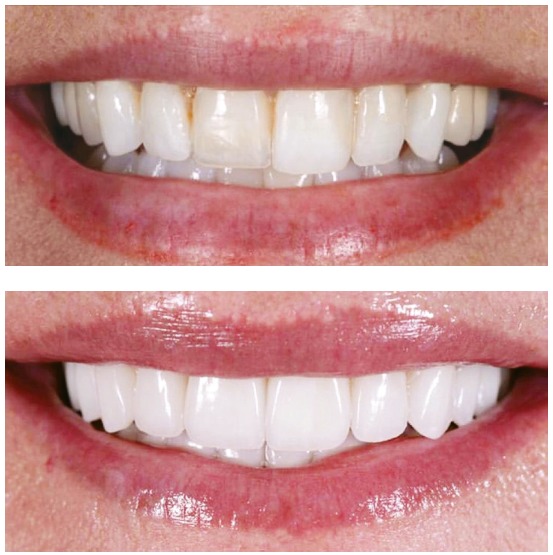
Pre- and post-treatment photographs of a case of whitening in which manipulation of final image is obvious, as even the patient's skin is whiter.

**Figure 4:**
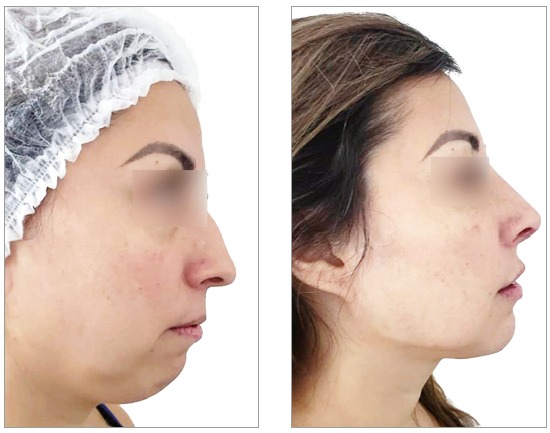
Pre- and post-treatment photographs of a facial harmonization case with head position manipulated in final photo to simulate profile improvement.

Despite the prohibition of use of pre- and post-treatment photographs, this practice has been widely prevalent. CROs should exercise control, start ethical confidential proceedings, grant dentists ample opportunity to defend themselves, and apply the appropriate penalties. Because of the flexibility and range of social media and networks, these procedures are technically complex and demand a large control structure. The permission to post photographs would make it practically impossible to control publications, check copyright and digital manipulations (Figs 1, 2 and 3) and detect photographic technique artifices (Fig 4). Again, we should raise questions about whether allowing publication is the best strategy. This practice, which has been abusively used, may induce patients to misinterpretations. Moreover, it produces consolidated case law that is unfavorable to the whole dental class. Alternatively, would it be better to improve control and guidance mechanisms for dentists and patients to discourage abuse and, therefore, raise the value of our profession?

## FINAL CONSIDERATIONS

The efficiency of social media and networks for interactions with patients and colleagues, as well as marketing resources and tools to disseminate and access scientific knowledge, is unquestionable^18^ ^,^ ^19^ ^,^ ^20^ ^,^ ^21^. To give up using them or to oppose their use would be anachronistic and unwise. Instead of blaming the tools, we should discuss and raise questions about their adequate use.

Several possible uses for online social media and networks do not break the law or disrespect ethical principles. Dentist may and should advertise their professional trajectories, fields of work and treatment procedures and techniques by focusing on information that effectively contributes to the understanding of the population in an educational manner that does not typify commodification, unfair competition or devaluation of the dental profession. 

Current postings have a predominant cosmetic appeal, stressing the magical comparisons of pre- and post-treatment appearance, of extra-white smiles in manipulated, expressionless faces. The indication of extensive esthetic procedures, very often for young patients, has become epidemic and completely disregards long-term effects. Dentistry seems to have abandoned its role as a healthcare profession. What we have seen in social networks is not health promotion.

In this context, we should not give up promoting the value of Dentistry. We cannot give up the defense of greater interests and the protection of our patients. For that purpose, the best advice comes from Ethics, which is independent of fads and always dynamic in the face of new dilemmas and challenges.

Ethics is a set of values and principles that enable the harmonious and respectful interactions between different people, ideas and ideologies in favor of the whole community, disregarding individualistic and deceptive interests. In everyday practice, when faced with ethical dilemmas, we should ask ourselves, Do I want it? Should I? Can I? While Want and Should may be subjective, Can is limited by the objectivity of ethical principles and legislation.

In some comfortable contexts, what I want is also what I should and can do. Unfortunately, many times there are things that I want, but should not do. There are things that I should do, but do not want to. In cases of conflict, all we can do is choose. After all, Ethics is a matter of choice^22^. Therefore, if you use online social media and networks unethically, you do not do it because you have to, because “everybody else is doing it”, or “the patients and the market demand it.” You do it because you choose so. Let us have the courage and the nobleness of always choosing the best for Dentistry.
